# Analyses of soluble endoglin and matrix metalloproteinase 14 using enzyme-linked immunosorbent assay in the diagnosis and assessment of severity of early- and late-onset pre-eclampsia

**DOI:** 10.4274/jtgga.galenos.2020.2019.0201

**Published:** 2021-02-24

**Authors:** Ali Ovayolu, Gamze Ovayolu, Erbil Karaman, Selver Güler, İlkay Doğan, Tuncay Yüce

**Affiliations:** 1 Clinic of Obstetrics and Gynecology, Cengiz Gökçek State Hospital, Gaziantep, Turkey; 2 Umay In Vitro Fertilization Center, Gaziantep, Turkey; 3 Department of Obstetrics and Gynecology, Van Yüzüncü Yıl University Faculty of Medicine, Van, Turkey; 4 Department of Public Health Nursing, Hasan Kalyoncu University Faculty of Nursing, Gaziantep, Turkey; 5 Department of Biostatistics, Gaziantep University Faculty of Medicine, Gaziantep, Turkey

**Keywords:** Endothelial dysfunction, hypertension, implantation, severe pre-eclampsia, trophoblast

## Abstract

**Objective:**

Abnormal trophoblastic invasion and impaired placentation have a crucial role in the etiopathogenesis of preeclampsia (PrE). Trophoblastic cells are involved in invading the maternal decidua and remodelling of the spiral arteries with matrix metalloproteinase-14 (MMP-14). MMP-14 cleavage of endoglin releases its extracellular region, the soluble form of endoglin (s-ENG), into the maternal circulation. In PrE, there is a relationship between endothelial dysfunction and s-ENG concentration. The aim was to determine and compare the serum levels of s-ENG and MMP-14 in different groups of PrE patients and healthy subjects.

**Material and Methods:**

The study included 30 patients with late-onset preeclampsia (L-PrE) (group 1; gestational age ≥34 weeks), 33 patients with normal pregnancy (group 2; gestational age ≥34 weeks), 31 patients early-onset preeclampsia (E-PrE) (group 3; gestational age <34 weeks), and 31 patients with normal pregnancy (group 4; gestational age <34 weeks). s-ENG and MMP-14 concentrations measured using enzyme-linked immunosorbent assays were compared.

**Results:**

In all groups, MMP-14 concentrations decreased with increasing gestational age. s-ENG concentrations were highest in the E-PrE group. In groups 1 and 3, 29 had mild PrE while 32 suffered severe PrE and s-ENG concentrations did not differ between mild and severe preeclampsia (p=0.133). However, there was a significant difference in MMP-14 concentration comparing mild with severe PrE (3.11±0.61 vs 3.54±1.00; p=0.047, respectively). There was no correlation between s-ENG and MMP-14 concentrations.

**Conclusion:**

MMP-14 and s-ENG concentrations can be predictive biomarkers for the diagnosis of PrE. Maternal serum MMP-14 concentration may be a biomarker for determining the severity of PrE.

## Introduction

Preeclampsia (PrE) remains an important obstetric syndrome and has a major effect on maternal/infant morbidity/mortality worldwide (1). The main features include protein leakage into urine and new-onset gestational hypertensive disease, or other signs/symptoms of PrE in the absence of proteinuria after 20 weeks’ gestation. Although improvements have been reported in the diagnosis and treatment of PrE in previous studies, there are still many unanswered questions. In PrE, the reduced capacity of extravillous trophoblasts (EVTs) for invasion into the spiral arteries results in an inadequately perfused fetal-placental unit. When the embryo is implanted, EVTs invade the decidua and remodel spiral arteries getting as deeply as the inner third of the myometrium. Matrix metalloproteinases (MMPs) enable the infiltration of EVTs into the uterine wall. Decidual stromal cells produce high concentrations of MMPs, enhancing the invasiveness of the EVTs (2,3). Dysregulation of MMPs causes inadequate trophoblast invasion, inadequate uterine and spiral artery remodeling, which may result in the development PrE (4,5).

MMPs are calcium- and zinc-dependent proteases that break down different components of the extracellular matrix (ECM). MMPs are key to the mediation of apoptosis, cell proliferation, cell-cell adhesion, cell migration and invasion, and tissue remodeling (6). The presence of MMP-14 has been demonstrated in the membrane of trophoblast and vascular endothelial cells (7). In normal pregnancy, there is a notable increase in MMP-14 concentration in the last trimester versus the first trimester (8). In PrE, the abnormal release of vasoactive factors such as MMP-1 and MMP-14 occurs near the end of pregnancy, thereby contributing to the development of hypertension. MMP-1 and MMP-14 have also been investigated in other obstetric syndromes, such as premature rupture of membranes (PROM) and preterm labor. The role of MMPs has been investigated in a wide range of conditions including inflammation and malignant growth, as well as reproductive and neurologic disorders (2). Further, analysis of gene expression demonstrated that both MMP-14 and endoglin gene expression was increased in PrE (9).

Endoglin (CD105) is an integral, membrane-bound glycoprotein and one of its functions is as a co-receptor for transforming growth factor-beta. High endoglin concentrations on the syncytiotrophoblasts in patients with severe PrE have been reported using western blot analysis and immunohistochemistry staining. A soluble form of endoglin (s-ENG) has been demonstrated in human blood. s-ENG is released from endothelial tissues, phagocytes, syncytiotrophoblasts, and smooth muscle cells. The role and direct molecular mechanism of s-ENG is not clear; however, it is capable of reducing angiogenesis (10,11). MMP-14 cleavage of endoglin releases s-ENG into the maternal circulation. The rise in s-ENG concentration in PrE is proportional to the severity of the disease and reduces after delivery. The relationship between endothelial dysfunction following poor placentation and s-ENG has also been shown in PrE. Several studies have shown that MMP-14 has importance in the reduction of s-ENG concentrations leading to alleviation of the clinical manifestations of PrE (2,8).

There is currently no mechanism to predict PrE or confirm PrE diagnosis before clinical occurrence. Many studies have investigated MMP-14 in PrE. However, there are no studies measuring serum MMP-14 concentrations by enzyme-linked immunosorbent assay (ELISA) in PrE. ELISA is a less-invasive, easy, fast, and inexpensive method. The aim was to evaluate the values of maternal serum MMP-14 and s-ENG in patients with PrE compared to healthy pregnancies, and to investigate the possible role of these biomarkers in assessment of PrE severity.

## Material and Methods

### Patient selection

**Figure 1:**
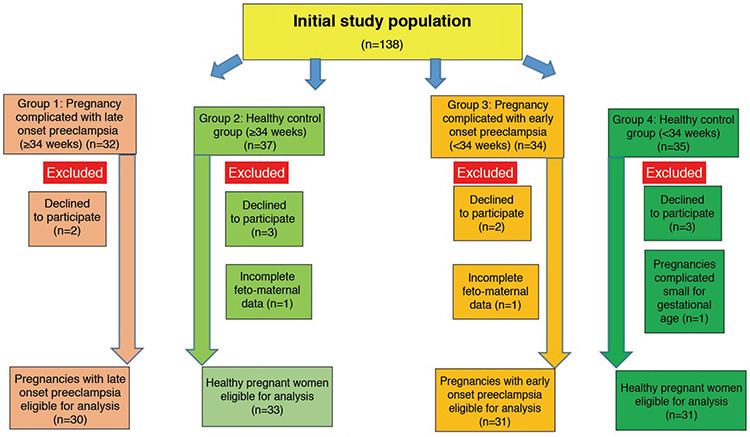
Flow chart of the pregnant women recruited in the study

Our subjects were prospectively recruited from the Clinic of Obstetrics and Gynecology at Cengiz Gökçek Public Hospital, Gaziantep, Turkey, between January 2018 and December 2018. This study was conducted according to the Declaration of Helsinki, and the Institutional Ethical Review Board of Gaziantep University Faculty of Medicine approved the study (approval number: 2018/91). A total of 138 pregnant women were recruited to the study, out of which 13 were excluded on grounds of incomplete fetomaternal details, refusal to participate in the study, and an SGA fetus in the control group (Figure 1). The remaining 125 women were divided into groups as follows. Thirty women with late-onset (≥34 weeks of gestation) preeclampsia (L-PrE) formed group 1; 33 healthy women at ≥34 weeks gestation were recruited to group 2; 31 women with early-onset (<34 weeks gestation) preeclampsia (E-PrE) formed group 3; and 31 healthy women at <34 weeks pregnancy were recruited to group 4. Groups 2 and 4 were matched for maternal and gestational age with groups 1 and 3, respectively. The control groups comprised women with healthy pregnancies who presented to our hospital for routine obstetric examination. All subjects were informed about the study and each gave written consent. Gestational age assessment was based on last menstrual period or first-trimester ultrasonographic obstetric measurements.

The diagnosis of PrE was based on the presence of proteinuria (urinary excretion of protein ≥300 mg in a 24-hour urine specimen, or proteinuria ≥1+ in dipstick) and a maternal blood pressure of ≥140/90 mmHg (a mean of two blood pressure measurements obtained six hours apart), occurring after 20 weeks of gestation in a previously normotensive woman, as defined by the Committee on Terminology of the American College of Obstetricians and Gynecologists. Mild PrE was defined as a blood pressure between ≥140/90 mmHg and 160/110 mmHg (for systolic and/or diastolic measurement) and if values exceeded 160/110 mmHg it was accepted as severe, as previously described (12). Small for gestational age (SGA) newborns were accepted as birth weight <10th percentile for gestational age with Turkey’s national nomogram as the reference for fetal growth (13). Maternal body mass index (BMI) was calculated in kg/m2 using the standard formula. Exclusion criteria for both groups were: pregnant women with any systemic disease, such as chronic hypertension, diabetes mellitus, thyroid diseases, liver and kidney diseases; use of any kind of medication throughout pregnancy; use of any medication for PrE treatment at the time of first admission; history of pregnancies complicated by PROM or chorioamnionitis; fever at the time of first admission; fetal congenital abnormalities or genetic syndromes; smoking during pregnancy; multiple gestation; and active labor.

Each pregnant woman had obstetric ultrasound examination and fetal/maternal assessment, which were conducted by a single obstetrician-gynecologist specialist (AO). Obstetric anamneses were obtained from all pregnant women. Demographic data, such as age, gravidity, parity, BMI, and gestational age were recorded. Maternal venous blood samples were taken for measurement of s-ENG and MMP-14 concentrations after the diagnosis of PrE in the outpatient clinic. The samples were immediately centrifuged at 1500 g for 10 min, and serum samples were separated and stored at -80 °C until required for analysis. All patients with E-PrE were hospitalized. After hospitalization, a betamethasone injection (12 mg) was administered without delay. Pregnancy was terminated immediately in the event of urgent fetal/maternal situations such as severe PrE development or fetal distress. Otherwise, maternal blood pressure was measured at least every 4 hours during rest with the arm held at the level of the heart. Hypertension can persist for short intervals in patients with diastolic and/or systolic blood pressure ≥110 mmHg and ≥160 mmHg, respectively, to facilitate timely anti-hypertensive treatment. In cases of E-PrE, delivery should be delayed for at least 48 hours if maternal and fetal status permit. During this period, betamethasone injections for lung maturation (two doses of 12 mg at 24-hour intervals) were administered. All patients with L-PrE were also hospitalized and their pregnancies were terminated. Women with uncomplicated pregnancies were randomly selected at the same time as the case selection was performed to serve as controls. The samples from the control groups were obtained during routine obstetric examinations in the last trimester of pregnancy. These women were then followed up until delivery. The four groups were compared in terms of maternal age, BMI, gravida, parity, week of gestation, systolic/diastolic blood pressure, full blood count, liver function tests including alanine aminotransferase and aspartate aminotransferase, blood urea nitrogen, creatinine, s-ENG, MMP-14 and total protein in spot urine sample, and infant weight at delivery.

### Serum MMP-14 and s-ENG analysis

MMP-14 concentrations were assessed using a commercial ELISA kit, the Human MMP-14 ELISA Kit (Rel Assay Diagnostics, Gaziantep, Turkey), in accordance with the manufacturer’s instructions. This ELISA kit (sensitivity range: 0.05 ng/mL; detection range: 0.1-30 ng/mL) is based on the principle of biotin double-antibody sandwich technique. The intra- and inter-assay variation coefficients were <8% and <10%, respectively. A commercial ELISA kit was also used for the assessment of s-ENG concentrations, specific for the detection of human s-ENG with high sensitivity and specificity (Rel Assay Diagnostics, Gaziantep, Turkey). The kit (sensitivity range: 0.23 ng/mL; detection range: 0.5-200 ng/mL) also used a sandwich-ELISA principle. The intra- and inter-assay variation coefficients were <8% and <10%, respectively.

### Statistical analysis

Statistical analyses were performed using SPSS for Windows, version 25.0, software package (IBM Inc., Armonk, NY, USA), and p values <0.05 were accepted as statistically significant. Results were presented as mean ± standard deviation (SD) after investigating normality distribution by Shapiro-Wilk test. In the analyses of the variables, the Student’s t-test was used for comparing two groups. ANOVA was used for the comparison of four groups. As a result of variance analysis, Tukey HSD test, one of the post-hoc tests, was used to determine the difference between the groups. In addition, the demographic data of the variables were investigated using frequency analyses.

## Results

**Figure 2:**
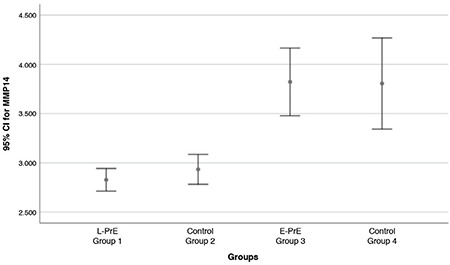
Error plot showing the mean value of matrix metalloproteinase-14 in the groups

A total of 61 pregnant women with PrE were included. The control groups included 64 healthy pregnant women. The demographic data of the groups were compared (Table 1). There was no significant difference between maternal age, gravidity, and parity, but there was a significant difference between the groups for BMI, gestational age, systolic blood pressure, diastolic blood pressure, and birth weights (p<0.05). The laboratory results of the study and control groups are shown in Table 2. MMP-14 and s-ENG concentrations differed between the groups, as shown in Table 2 and Figure 2, 3. MMP-14 concentrations did not differ between group 1 and group 2, nor did they differ between group 3 and group 4. Thus, MMP-14 concentrations were the same in women with PrE and healthy pregnancies matched for gestational age but did differ between earlier and later pregnancies, that is between <34 weeks and ≥34 weeks gestation. In all pregnant women, as the gestational age increased, MMP-14 concentrations in maternal serum decreased.

The highest concentrations of s-ENG were found in the E-PrE group. No differences were detected between s-ENG and MMP-14 concentrations in mild (n=21) and severe PrE (n=9) in the L-PrE group (p=0.829, p=0.210, respectively). In addition, no differences were detected between s-ENG and MMP-14 concentrations in mild (n=8) and severe PrE (n=23) in the E-PrE group (p=0.887, p=0.739, respectively). When early and late PrE groups (group 1 and group 3) were compared, no differences were detected between s-ENG concentrations in mild (n=29) and severe PrE (n=32) (p=0.133). However, there was a significant difference in MMP-14 concentrations in pregnancies affected by mild or severe PrE (3.11±0.61 vs 3.54±1.00, p=0.047, respectively) (Table 3).

When the patients with (n=10) and without (n=115) SGA infants were compared, no differences were found in s-ENG and MMP-14 concentrations (p=0.133, p=0.969, respectively). When the patients with (n=15) and without (n=110) new-onset cerebral or visual disturbances were compared, there were again no differences in s-ENG and MMP-14 concentrations (p=0.528, p=0.573, respectively). When the patients with (n=8) and without (n=117) right upper quadrant or epigastric pain were compared, no difference was found in s-ENG and MMP-14 concentrations (p=0.162, p=0.154, respectively). When those who had first gravidity (31 patients) and gravidity >1 (94 patients) were compared, no differences were found between the s-ENG and MMP-14 concentrations (p=0.855, p=0.364, respectively). No difference was found in serum concentrations of s-ENG and MMP-14 when subjects were compared in terms of BMI [<30 kg/m2 (n=56) and ≥30 kg/m2 (n=69)] p=0.373 and p=0.873 for s-ENG and MMP-14, respectively or for maternal age [<35 years (n=100) and ≥35 years (n=25)] p=0.167 and p=0.625 for s-ENG and MMP-14 respectively. No statistically significant correlation was detected between s-ENG and MMP-14 concentrations (p>0.05).

## Discussion

In the present study, maternal blood concentrations of s-ENG and MMP-14 were evaluated in order to examine the association between diagnoses of L-PrE/E-PrE, the severity of PrE, and these biomarkers. It was found that serum s-ENG and MMP-14 concentrations differed significantly between the two PrE groups and their respective matched control groups. The concentrations of s-ENG were at the highest in the E-PrE group (group 3) in which severe PrE was present. MMP-14 concentrations, on the other hand, were found to be highest in severe PrE cases.

The contact between the placenta and the decidua ensures metabolic exchange between the fetus and the mother. Trophoblast cells of the embryo initially attach to the uterine epithelium. Trophoblasts differentiate into EVTs, which degrade the uterine epithelium basement membrane and ECM, then migrate into the decidual stroma. EVTs are characterized by their invasiveness, ensuring sufficient contact with the maternal circulation. Unsurprisingly, this invasion stage is under strict biological control and restricted to the decidua and the proximal third of the myometrium in healthy pregnancies. When this invasive process is dysregulated fetal growth restriction and PrE may result. Invasive processes are generally assisted by the expression and activity of MMPs (8). EVTs also reach and remodel the spiral arteries, transforming them into low resistance vessels, a process necessary to allow an adequate blood supply to the fetus. It has been shown that this process is regulated by MMP-14 and MMP-15, mediated by endothelin-1 in a low oxygen environment (2-3% O_2_) (14). These pathologic changes constitute a process that starts in the first trimester, proceeds through a normal pregnancy period, and are finally reflected in an excessive clinical manifestation in the last trimester. These events consist of pathogenic factors that are produced in the placenta as a response to hypoxia, mixing with the maternal blood and causing endothelial dysfunction. Despite the presence of abnormally high concentrations of excreted molecules, inflammatory factors, autoimmunity or anti-angiogenic processes are far more important (15).

Several authors have demonstrated that the ECM and MMP-14 were vital regulators of angiogenesis (16,17). Placental MMPs may influence spiral artery remodeling in implantation. In PrE, MMPs have roles in prompting vasoconstriction, changes in vascular reactivity, and pathological damage to the endothelium. Thus, there has been an increase in interest in the pathological effect of the imbalance of angiogenic and/or anti-angiogenic factors in PrE, including endoglin/s-ENG and MMP-14. The close association between the location and expression levels of MMP-14 and the same features relating to endoglin has attracted much interest. MMPs have become important biomarkers for the identification of women with an elevated risk of developing PrE and also important biologic targets for treating this syndrome (1,7,18). The process of investigating MMP-14 tissue level and expression postpartum is complex, laborious and expensive. The aim of this study was to evaluate MMP-14 through direct measurements in maternal serum, a minimally invasive process, which can be repeated throughout pregnancy. The drawback of serum measurements of MMP-14 are that these will not reflect tissue specific expression patterns in normal or PrE pregnancy, because of the significant membrane-binding properties of MMP-14 (19).

Zhang et al. (7) found that s-ENG concentrations were high in patients with severe PrE, and showed that this event was triggered by MMP-14, leading to the development of severe PrE. In contrast, Levine et al. (9) suggested that there were no important differences in s-ENG concentrations between women with mild PrE and severe PrE, reporting no correlation between s-ENG concentrations and the severity of PrE. However, it was also shown that there was an evident increase in s-ENG concentration, starting 2-3 months before the onset of PrE. They also reported that this increase was greater in E-PrE than in L-PrE (9,20). Sezer et al. (4) conducted a study in a PrE group comprising patients with E-PrE and L-PrE, and found that s-ENG concentrations in PrE were high in the maternal circulation and also umbilical cord blood. However, there was no difference between both preeclamptic groups regarding s-ENG concentration of the maternal circulation (4). Zafer et al. (11) compared s-ENG concentrations in maternal serum and amniotic fluid and reported that they were incompatible. All these findings suggest that all compartments have independent dynamics in terms of s-ENG. Moreover, it is possible that MMP-14 may have independent dynamics, because of the close biological association with s-ENG. When all patients with severe PrE were analyzed, MMP-14 concentrations were found to be higher although s-ENG was not different. Interestingly, this might suggest that MMP-14 serum concentrations in PrE may be more significant than s-ENG. Although s-ENG and MMP-14 concentrations showed a similar pattern, the relationship between the two showed no statistically significant correlation. This may be because of the membrane-binding properties of MMP-14.

### Study Limitation

We acknowledge strengths and limitations of the study. The most important limitation of our study was the small number of participants. We do not have the pre-pregnancy BMI of all participants. s-ENG and MMP-14 concentrations could have been examined in different compartments, such as the placenta, umbilical cord serum, and amniotic fluid. The other limitation was that there are no uterine artery Doppler studies in this study. The strengths of the study were that none of the patients had any treatment for PrE and only participants who were not in active labor were selected for the study. Another strength was the comparison of MMP-14 serum concentrations with s-ENG, which has a major role in angiogenesis and endothelial cell function.

## Conclusion

Larger basic and clinical studies are necessary to assess whether MMPs have an important role in the pathophysiology of PrE. In addition, meta-analyses should be performed to investigate the predictive value of these enzymes as biomarkers or therapeutic targets. Finally, in view of the literature data, we believe that testing s-ENG is worthy of further investigation for the early diagnosis of PrE. In addition, it appears reasonable to investigate serum MMP-14 concentrations in predicting patients who will proceed to a severe form of PrE.

## Figures and Tables

**Table 1:**
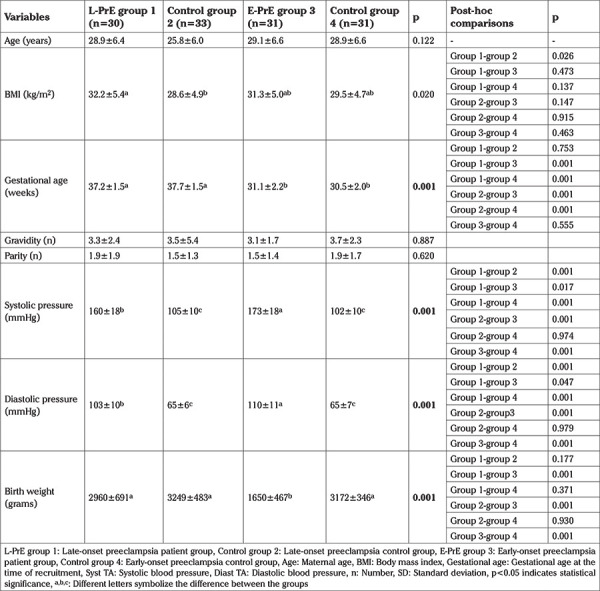
The demographic data of the groups

**Table 2:**
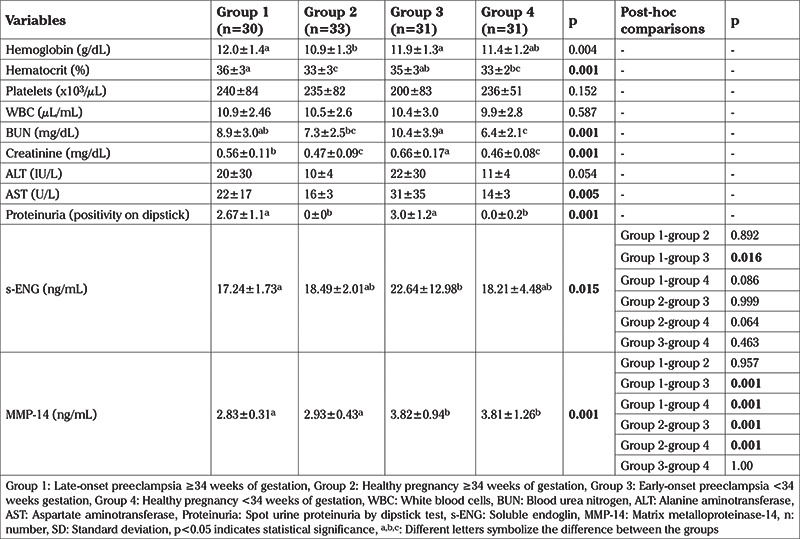
The laboratory parameters of the four groups

**Table 3:**
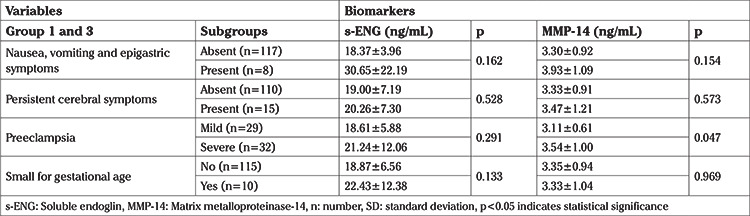
Clinical characteristics and biomarkers levels in the preeclampsia groups, group 1 (late PrE) and group 3 (early PrE). Biomarker data is given as mean ± SD

**Figure 3:**
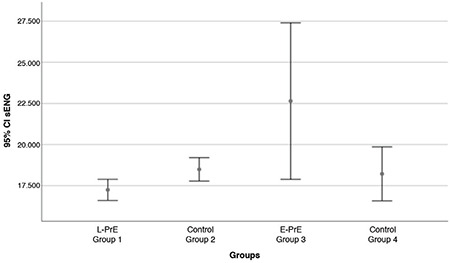
Error plot showing the mean value of soluble endoglin in the groups
